# Fermentation-Generated Wet Foams: Working-Volume Scaling, Temporal Evolution and Nominal-Freeboard Burden

**DOI:** 10.3390/ma19214755

**Published:** 2026-11-09

**Authors:** Angelos T. Zamanis, Sotiris P. Evgenidis, Thodoris D. Karapantsios, Margaritis Kostoglou

**Affiliations:** Lab of Chemical and Environmental Technology, School of Chemistry, Aristotle University of Thessaloniki, 54124 Thessaloniki, Greece; sevgenid@chem.auth.gr (S.P.E.); karapant@chem.auth.gr (T.D.K.); kostoglu@chem.auth.gr (M.K.)

**Keywords:** wet foam, alcoholic fermentation, wine must, apparent foam volume, foam persistence, freeboard, vessel utilization

## Abstract

Foam generated during wine-must fermentation can reduce the vessel space available for processing liquid. Its practical impact depends on the amount of foam, its persistence, and its position relative to the initial liquid level. This study quantified these features in five must batches: Sauvignon Blanc, Vidiano, Xinomavro rosé, Syrah rosé, and a 50:50 Syrah/Cabernet Sauvignon co-vinification. Two types of experiments used transparent open-topped 1 L vessels. The first tested four initial liquid volumes (500, 600, 700, and 800 mL), with three independently fermented vessels per batch–volume combination (60 vessels), at approximately 12 °C for white musts and 14 °C for rosé musts. The second followed three vessels per batch containing 700 mL at a common temperature of 12.0 ± 0.3 °C, with 12 sampling times from day 0 (D0) to day 16 (D16). Photographic measurements estimated apparent foam volume and recorded the underlying bulk-liquid level. Peak foam volume increased by 11.51–14.16 mL per additional 100 mL of must; peak foam-to-initial-liquid ratios were 11.75–13.29% across batch means. Foam reached sampled maxima on days 10–11 and remained visible at D16. Area under the foam-volume curve ranged from 798.60 ± 1.89 to 954.85 ± 8.89 mL·day. At 800 mL initial fill, peak apparent foam corresponded to 46.83–53.17% of the initial 200 mL headspace. Paired data at 700 mL showed that the lower foam boundary moved downward during foam development and partly recovered as foam declined; total foam-layer burden is therefore distinct from net protrusion above the initial liquid level. These batch- and vessel-specific results support preliminary assessment of fermentation foam and freeboard, but do not establish general varietal rankings or industrial safe-fill limits.

## 1. Introduction

Foam is an operationally important response during alcoholic fermentation because it occupies gas-disengagement space and can limit usable vessel capacity. The International Organisation of Vine and Wine (OIV) identifies foam management as a means of preventing overflow losses and improving cellar-capacity utilization [1]. Because freeboard reserved for transient foam is installed capacity unavailable for liquid working volume, its prediction or control may also affect tank utilization and capacity-related investment requirements. Fermentation studies have likewise shown that foam accumulation depends on process conditions and may vary spatially within the foam phase [2]. A process-relevant description therefore requires both the magnitude of the foam layer and its evolution through time. From a soft-matter perspective, fermentation-generated foam is a dynamically produced wet foam whose transient structure links materials behavior with process-engineering constraints.

The fermenting matrix and the inoculated yeast can both influence foam formation and persistence. A foam-promoting mannoprotein precursor has been identified in an oenological Saccharomyces cerevisiae strain [3], while wine-related studies have associated proteins, amino acids, polysaccharides and other macromolecular constituents with foamability and foam stability [4,5,6]. Yeast-screening studies likewise document strain-dependent technological traits [7,8,9], while the OIV characterization guidelines include foam production among the technological properties used to describe wine yeasts [10]. Together, these observations support a defined inoculation protocol while matrix and kinetic effects remain possible.

At the physical level, wet-foam behavior reflects bubble packing, interfacial area, liquid redistribution, drainage, coarsening and film rupture [11,12,13,14,15,16,17]. Container diameter, wall wettability and vessel shape can alter drainage [18], and the food-foam literature identifies naturally occurring surface-active compounds, particularly proteins, as important determinants of formation and stability [19,20,21,22]. In a fermenting vessel, these mechanisms act within a foam layer that is continuously supplied with carbon dioxide, so apparent foam volume represents the combined outcome of gas generation and retention rather than a direct measurement of either process alone.

Wine-foam studies further emphasize the chemical complexity of the matrix. Proteins, glycoproteins and interactions among wine constituents can influence foam expansion and persistence [23,24,25], while yeast-derived proteins and cell-wall material can also contribute [3,26,27]. However, compositional mechanisms can only be inferred when those constituents are measured independently. In the present work, the five systems are therefore treated as the five specific must batches studied, not as replicated representatives of grape varieties.

The present study addresses a methodological gap by combining, within a single vessel-resolved framework, working-volume scaling, longitudinal persistence quantified by area under the curve (AUC), paired lower-interface displacement and nominal-freeboard burden. It therefore addresses two questions: First, how does peak apparent foam burden scale with initial working volume under the practical temperature regime applied to each batch? And second, how do foam trajectories and persistence differ among the same batches at a common temperature? Peak apparent foam volume, the liquid-normalized ratio *α*, vessel-level time-integrated accumulation and a nominal-freeboard burden index were used jointly to describe these responses. Paired bulk-liquid measurements distinguished apparent foam-layer burden from net protrusion above theinitial liquid level, while projected contour profiles provided a descriptive spatial complement to the scalar endpoints.

## 2. Materials and Methods

### 2.1. Must Batches, Inoculation and Experimental Design

Five must batches were investigated: Sauvignon Blanc, Vidiano, Xinomavro rosé, Syrah rosé, and a 50:50 Syrah/Cabernet Sauvignon co-vinification. Their naturally occurring initial soluble-solids contents were approximately 22 °Brix. No sugar addition, dilution, or other adjustment of soluble-solids content was performed prior to inoculation. Here °Brix refers to soluble solids expressed as sucrose equivalents and not to a direct analytical determination of glucose or fructose. Vitilevure B + C (Martin Vialatte Œnologie, Magenta, France; purchased from Ampelooeniki, Thessaloniki, Greece) inoculum was added at 0.20 g·L^−1^ following rehydration.

Fermentation was carried out in transparent, graduated, open-topped cylindrical DURAN vessels (DWK Life Sciences GmbH, Wertheim, Germany; nominal capacity *V*_T_ = 1000 mL; reported internal diameter *d* = 100 mm). The tops remained open during fermentation; no fermentation funnel, airlock, stopper, or other gas-release fitting was used. Evolved CO_2_ was therefore released directly into the laboratory atmosphere under non-pressurized conditions. No mechanical agitation was applied after inoculation.

The working-volume experiment comprised four initial liquid charges (500, 600, 700, and 800 mL) in three separately fermented vessels per batch–charge combination (5 × 4 × 3 = 60 vessels). Sauvignon Blanc and Vidiano were maintained at approximately 12 °C, while the rosé systems were maintained at approximately 14 °C. Each system originated from one homogenized must batch. The vessel was the experimental unit; the three vessels do not represent independent harvest or variety replicates. Because temperature differed between white and rosé systems, working-volume system effects characterize the joint batch/temperature condition, not an isolated matrix effect.

A separate controlled longitudinal experiment used three individually tracked 700 mL vessels for each batch at a common temperature of 12.0 ± 0.3 °C. Sampling occurred on D0, D3, D5, D7, D8, D9, D10, D11, D12, D14, D15, and D16 (D denotes fermentation day), giving 15 vessels, 180 foam observations, and 180 paired foam–bulk-liquid readings. Measurements ended on D16. Afterwards, active temperature control was discontinued, and the must temperature was allowed to approach ambient laboratory temperature to encourage the continuation of alcoholic fermentation and reduce the risk of sluggish or incomplete sugar conversion. No post-D16 observations are included in the controlled dataset. Inference concerns these five specific must batches and not general varietal rankings.

### 2.2. Apparent Foam and Bulk-Liquid Measurements

**Figure 1:**
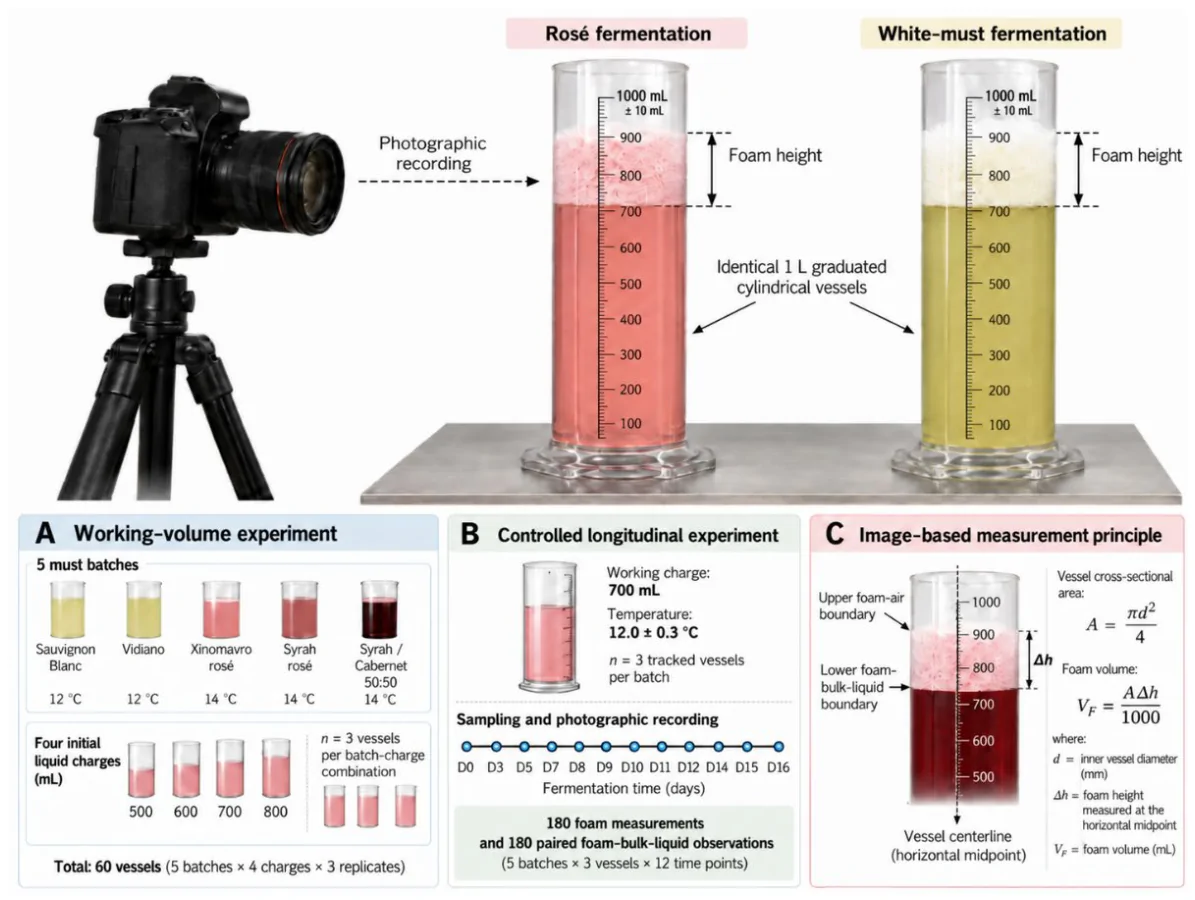
An illustration of photographic monitoring, the 60-vessel working-volume experiment, the 15-vessel controlled longitudinal experiment, and the image-based foam measurement principle. The illustration is schematic and is not an archival photograph of the actual experiment.

Photographs were acquired using a Canon EOS 70D digital camera (20.2 megapixels) with a Canon EF 100 mm f/2.8 Macro lens (Canon Inc., Tokyo, Japan). Time-stamped photographs and vessel graduations were used to characterize the visible wet-foam layer (Figure 1). Images were inspected in CorelDRAW Graphics Suite 2024 (version 25.2; Corel Corporation, Ottawa, ON, Canada), and contour coordinates were digitized in TechDig (version 2.0; Ronald B. Jones, Mundelein, IL, USA) when necessary. The apparent foam layer was bounded by its upper foam–air interface and the lower boundary of the visually continuous bulk-liquid phase. For scalar calculations, foam height *Δh* was measured at approximately the horizontal midpoint of the visible layer; bulk-liquid height was measured from the vessel base to the lower foam boundary. The reported peaks represent the largest sampled readings, not continuous-time maxima. In the 60-vessel working-volume series, each reported vessel peak denotes the greatest available time-stamped photographic reading. The frequency of image acquisition for that series was not documented in the records retained for this analysis; these values must therefore be interpreted as sampled maxima, not continuously monitored absolute peaks.

The cross-sectional area of a cylindrical vessel was calculated as *A* = π*d*^2^/4. Apparent foam volume was calculated from *V*_F_ = *AΔh*/1000, with *A* in mm^2^ and *Δh* in mm. At the reported internal diameter of 100 mm, *A* = 7853.98 mm^2^ and 1 mm of height corresponds to 7.854 mL; a 10 mL graduation interval corresponds to 1.273 mm. Auxiliary readings from a Mitutoyo ABSOLUTE digital caliper (model 500-196-30; Mitutoyo Corporation, Kawasaki, Japan; nominal resolution 0.01 mm) were used in image inspection. All diameter-dependent height-to-volume conversions and the sensitivity estimates below are conditional on the reported 100 mm internal diameter; an independent calibration record was not available for retrospective verification.

As a transparent sensitivity check, uncertainties of 0.1, 0.5, and 1.0 mm in apparent foam height correspond geometrically to approximately 0.79, 3.93, and 7.85 mL of apparent foam volume, respectively. These are sensitivity equivalents, not empirically established repeatability values. Independent repeat-digitization measurements were not available. Accordingly, the small sample standard deviations across vessels describe observed between-vessel dispersion, not validated sub-milliliter image-reading accuracy.

Because the lower foam interface moves during fermentation, the apparent foam layer may extend below the initial liquid level. Its total apparent volume must therefore be distinguished from its net protrusion above the initial liquid level. This distinction was assessed using the paired bulk-liquid readings in the controlled 700 mL experiment.

As a descriptive spatial complement, projected foam profiles were digitized at D0, D5, D10, and D16. Local thickness *H*_F_(*x*,*t*) was normalized by its spatial mean, giving *η*(*x*,*t*) = *H*_F_(*x*,*t*)/${\bar{H}}_{F}$(*t*) over the nominal vessel diameter. Since replicate-level uncertainty for spatial contours was not determined, these profiles were interpreted descriptively without inferential between-batch comparisons.

### 2.3. Normalized Foam Response, Nominal-Freeboard Burden and Integrated Accumulation

The liquid-normalized peak apparent foam response (α) expresses the sampled maximum apparent foam volume relative to the initial must charge:

(1) $$\alpha =\frac{V_{F,max}}{V_{L,0}}$$

where *V*_F,max_ is the sampled peak apparent foam volume and *V*_L,0_ is the initial liquid charge. The nominal fill fraction (φ) is defined by:

(2) $$\varphi =\frac{V_{L,0}}{V_{T}}$$

where *V*_T_ is the nominal total vessel capacity. The apparent nominal-freeboard burden (β) relates the entire apparent foam layer at its sampled peak to the initially unoccupied vessel volume:

(3) $$\beta =\frac{V_{F,max}}{V_{T}-V_{L,0}}=\frac{\alpha \varphi }{1-\varphi }$$

Thus β is a foam-layer burden index; it is not necessarily the fraction physically located above the initial liquid level. Calculations for the working-volume experiment were confined to the tested fill fractions φ = 0.50–0.80.

In the controlled 700 mL experiment, the contemporaneous apparent bulk-liquid deficit was:

(4) $$D_{L}\left(t\right)=V_{L,0}-V_{L}\left(t\right)$$

where *V*_L_(t) is the image-derived bulk-liquid reading. The net nominal-freeboard intrusion fraction (γ) was then calculated as:

(5) $$\gamma \left(t\right)=\frac{V_{F}\left(t\right)-D_{L}\left(t\right)}{V_{T}-V_{L,0}}$$

This geometrically derived descriptor estimates foam protrusion above the initial liquid level by accounting for displacement of the foam–bulk boundary. *β* describes the whole apparent foam layer relative to the initial headspace, while *γ* describes its net intrusion. The corresponding volume-based geometric construction is shown in Supplementary Figure S1, using pooled D11 values solely as an illustrative example.

Cumulative apparent foam accumulation for each tracked vessel was determined over D0–D16 by trapezoidal integration using the actual, unequal sampling intervals:

(6) $$AUC=\sum_{i}\left[\frac{V_{F}\left(t_{i}\right)+V_{F}\left(t_{i+1}\right)}{2}\right]\times \left(t_{i+1}-t_{i}\right)$$

The area under the curve (AUC) has units of mL·day and combines foam magnitude with persistence. Vessel-level AUC values are reported as mean ± sample standard deviation (SD) for each batch. The time to sampled maximum is the measured day at which each vessel reached its greatest recorded foam volume.

### 2.4. Statistical Analysis and Descriptive Trajectory Fitting

Peak-volume responses were analyzed by ordinary least squares (OLS), with system as a categorical factor and initial working volume as a continuous predictor. The system × charge interaction was tested against an additive model; HC3 heteroscedasticity-consistent covariance was used for sensitivity analysis. Additive terms were assessed with partial F tests. Separate system-specific regressions yielded charge slopes and 95% confidence intervals (CI). Because the white and rosé series were run at different temperatures, the system term represents the combined operating condition rather than a temperature-independent effect of must composition.

The controlled trajectories were assessed using mixed-design repeated-measures analysis of variance (ANOVA), with system between vessels, sampling day within vessel, and vessel nested within system. Greenhouse–Geisser adjustment was applied to time-related degrees of freedom (*ε* = 0.2503). The model preserved the 15 individual tracked vessel identities. Vessel-level AUC was compared with one-way ANOVA followed by Tukey honestly significant difference (HSD) comparisons; a median-centered Levene test examined variance consistency.

The 180 paired foam–bulk observations were additionally analyzed by OLS for apparent bulk-liquid deficit, with apparent foam volume, system, and sampling day as predictors. Confidence intervals used vessel-clustered covariance, a small-sample adjustment, and a t reference distribution with 14 degrees of freedom. Since both quantities are defined using the lower foam boundary, the association is interpreted geometrically rather than as a liquid mass balance.

For descriptive visualization, a Gaussian envelope was fitted by nonlinear least squares to the five controlled system mean trajectories:

(7) $$V_{F,s}\left(t\right)=B_{s}+A_{s}\exp\left[-\frac{{\left(t-t_{p}\right)}^{2}}{2\sigma_{t}^{2}}\right]$$

The Gaussian was chosen because the observed trajectories were broadly unimodal, with an initial rise, a sampled maximum around D10–D11, and a subsequent decline. It provides a compact empirical summary through a system-specific baseline *B_s_* and amplitude *A_s_*, with shared fitted peak time *t*_p_ and temporal width *σ*_t_. It is not a mechanistic description of sugar-consumption kinetics, CO_2_ generation, drainage, or bubble rupture, nor does the high fit quality establish forecasting accuracy outside D0–D16. Goodness of fit was evaluated using pooled coefficient of determination (R^2^) and root mean square error (RMSE) across the 60 system–day mean observations. Computations used Python 3.12.7, NumPy 2.1.3, and SciPy 1.14.1, with two-sided tests at 0.05.

## 3. Results

### 3.1. Working-Volume Dependence of Peak Apparent Foam Volume

**Figure 2:**
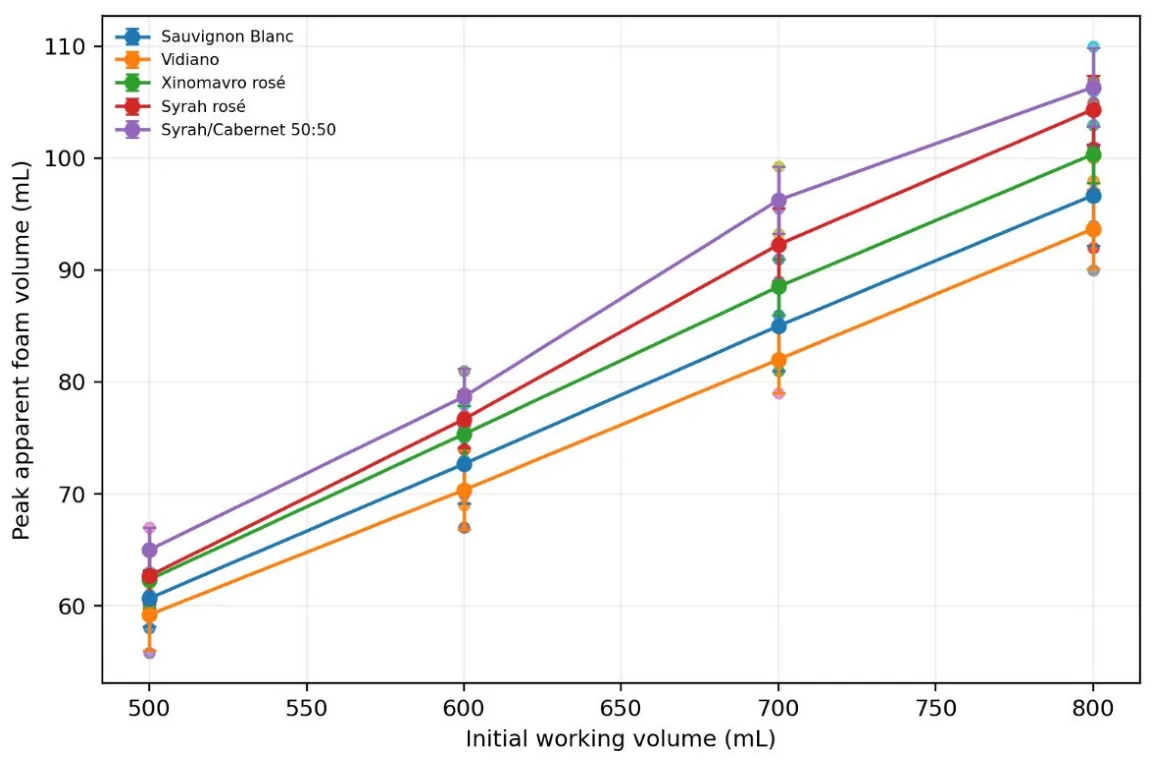
Peak apparent foam volume at four initial working volumes. Points show individual vessel readings; connected markers and error bars show condition mean ± sample SD.

Mean peak apparent foam volume increased with initial liquid charge in every operational system (Figure 2). Across 500–800 mL, the increase per additional 100 mL was 12.03 mL for Sauvignon Blanc, 11.51 mL for Vidiano, 12.72 mL for Xinomavro rosé, 14.06 mL for Syrah rosé and 14.16 mL for the Syrah/Cabernet blend. System-specific regression R^2^ values ranged from 0.952 to 0.979. Table 1 summarizes the loading slopes and liquid-normalized response; individual vessel measurements are given in Appendix A.

The additive model identified a strong working-volume effect on peak apparent foam volume, F(1,54) = 1348.46, *p* < 0.001, and a categorical system effect, F(4,54) = 21.54, *p* < 0.001. The conventional system × charge interaction was borderline, F(4,50) = 2.558, *p* = 0.04995, whereas HC3-robust inference did not support the interaction, F(4,50) = 1.861, *p* = 0.132. For *α*, the system effect was significant, F(4,54) = 22.78, *p* < 0.001, but neither the working-volume effect, F(1,54) = 1.606, *p* = 0.211, nor the interaction, F(4,50) = 0.793, *p* = 0.536, was significant. Thus, absolute apparent foam burden increased strongly with initial charge, while the liquid-normalized response remained approximately stable across the tested working-volume range. Because temperature differed between white and rosé batches in this experiment, the categorical system effect should not be interpreted as an isolated matrix or varietal effect.

### 3.2. Controlled Foam Trajectories and Integrated Accumulation

**Figure 3:**
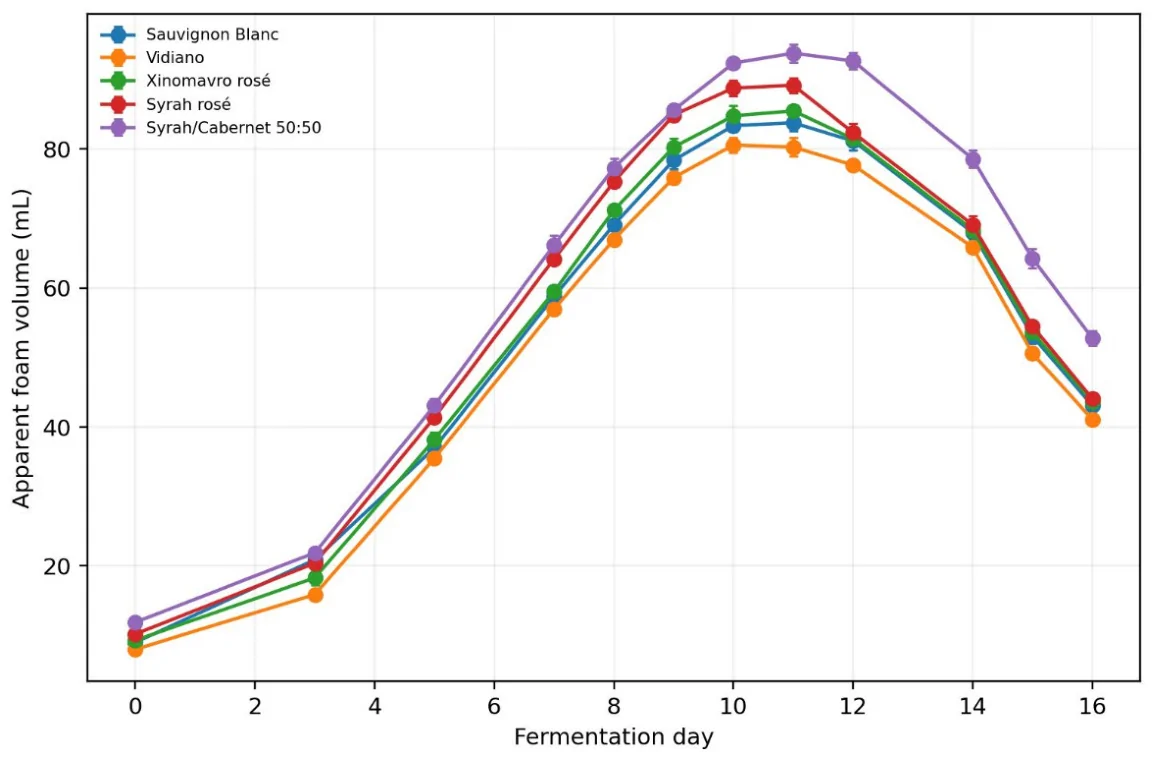
Controlled 700 mL apparentfoam trajectories at 12.0 ± 0.3 °C. The markers show the mean of three tracked vessels and the very narrow error bars show day-specific sample SD.

**Figure 4:**
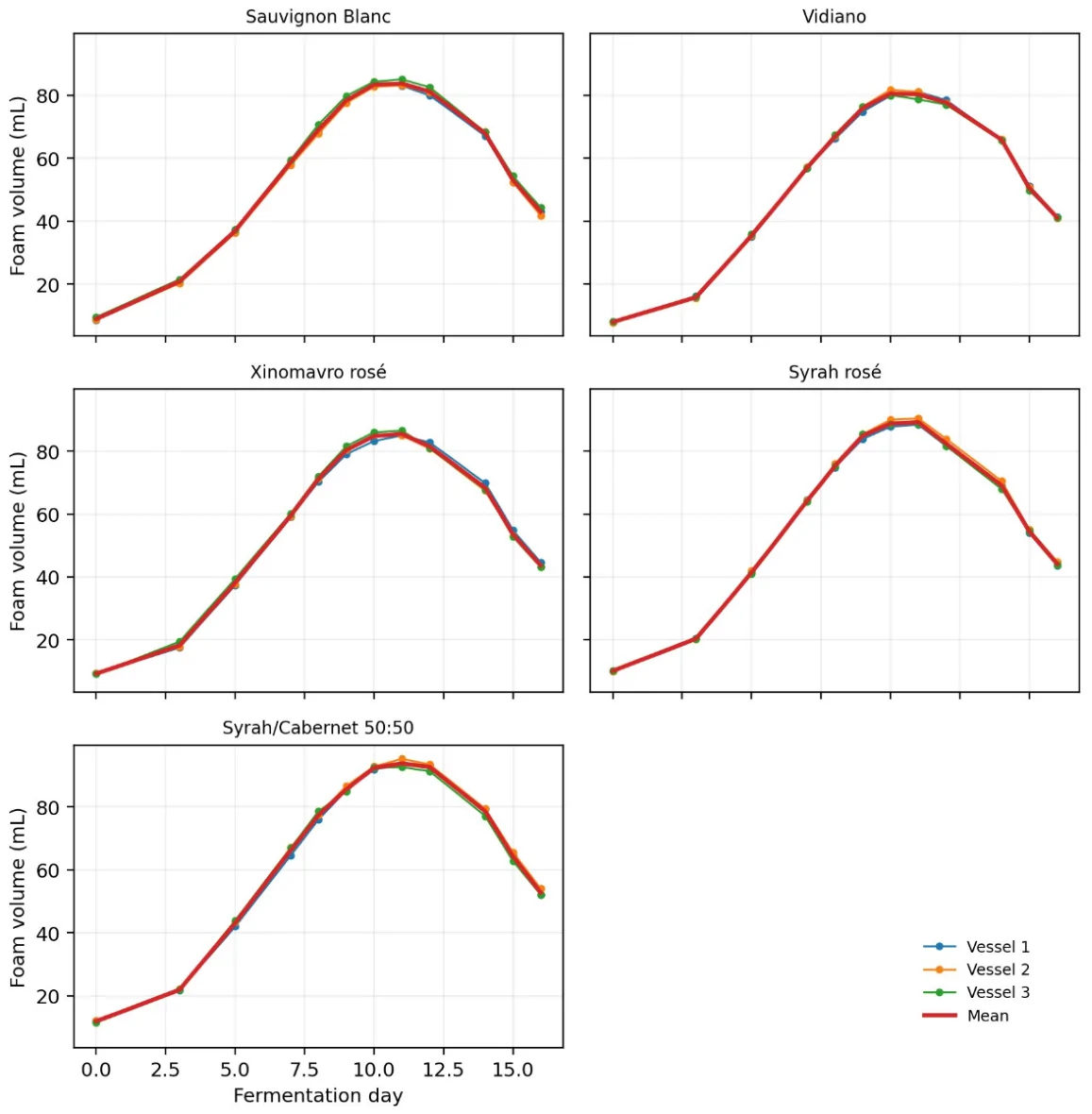
The individual apparentfoam trajectories for the three longitudinal vessels in each system. The heavier curve shows the corresponding system mean.

At the common 12.0 ± 0.3 °C regime, apparent foam volume increased through D10–D11 and then declined while remaining visible at D16 (Figure 3 and Figure 4). System mean sampled maxima (Table 2) were 83.8 mL for Sauvignon Blanc, 80.6 mL for Vidiano, 85.5 mL for Xinomavro rosé, 89.2 mL for Syrah rosé and 93.8 mL for the blend. The three Vidiano vessels reached their individual sampled maxima at D10 or D11; the remaining systems reached their sampled maxima at D11. Vessel-resolved measurements are given in Appendix B.

**Figure 5:**
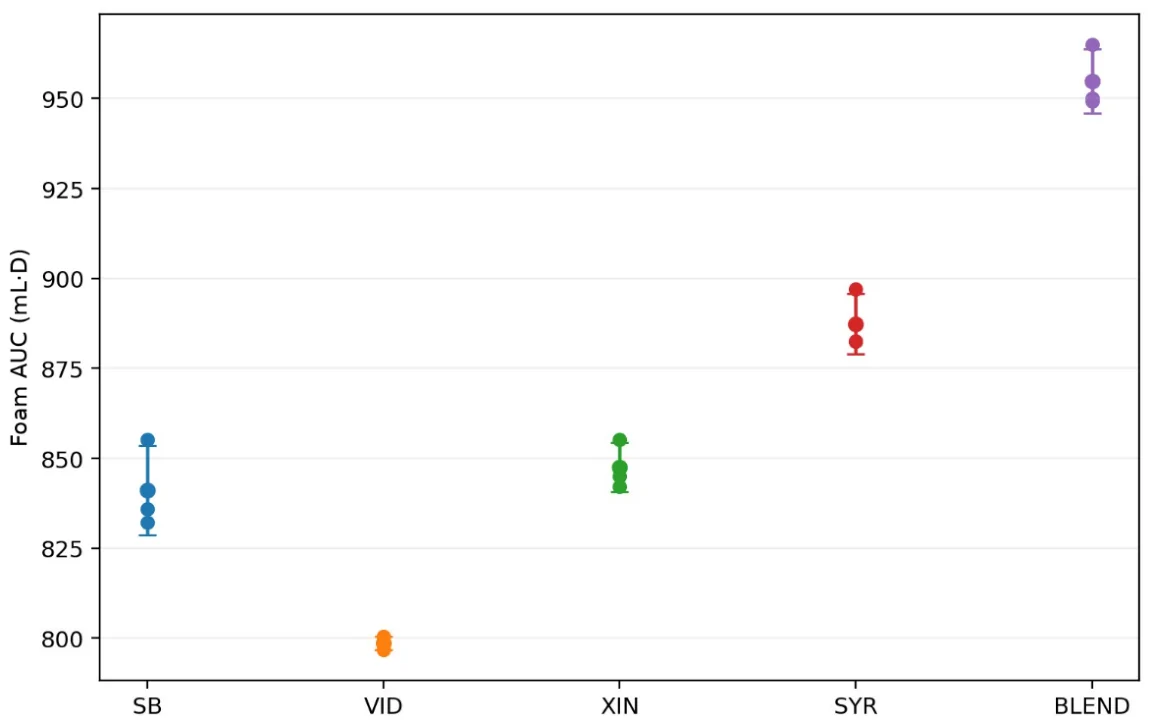
The individual vessel AUC values for the controlled 700 mL experiment. The larger markers and error bars show the system mean ± sample SD.

Vessel-level AUC (Figure 5) ranged from 798.60 ± 1.89 mL·D for Vidiano to 954.85 ± 8.89 mL·D for the blend. One-way ANOVA identified differences among the five investigated batches, F(4,10) = 147.33, *p* < 0.001; the median-centered Levene statistic was W = 0.291, *p* = 0.877. Tukey comparisons placed the blend above all other batches and Syrah rosé above Sauvignon Blanc, Vidiano and Xinomavro rosé. Sauvignon Blanc and Xinomavro rosé did not differ (adjusted *p* = 0.881), and each exceeded Vidiano. The blend AUC was 19.57% greater than the Vidiano mean. These rankings apply to the specific must batches tested at the common 12 °C regime.

The repeated-measures analysis identified system, day and system × day effects (Table 3). The interaction indicates that the trajectories were not identical across the five batches, even though all shared a broad rise-and-decline pattern.

### 3.3. Descriptive Gaussian Envelope

The shared-shape Gaussian envelope described the controlled system means with R^2^ = 0.9960 and RMSE = 1.595 mL. The fitted shared peak-time parameter was *t*_p_ = 10.804 D and the width was *σ*_t_ = 4.318 D. System-specific baseline and amplitude coefficients are given in Table 4. This fit summarizes the dominant temporal pattern; the significant system × day effect from the repeated-measures analysis shows that smaller system-dependent trajectory differences remain despite the high pooled fit.

### 3.4. Apparent Foam and Bulk-Liquid Interface Changes

**Figure 6:**
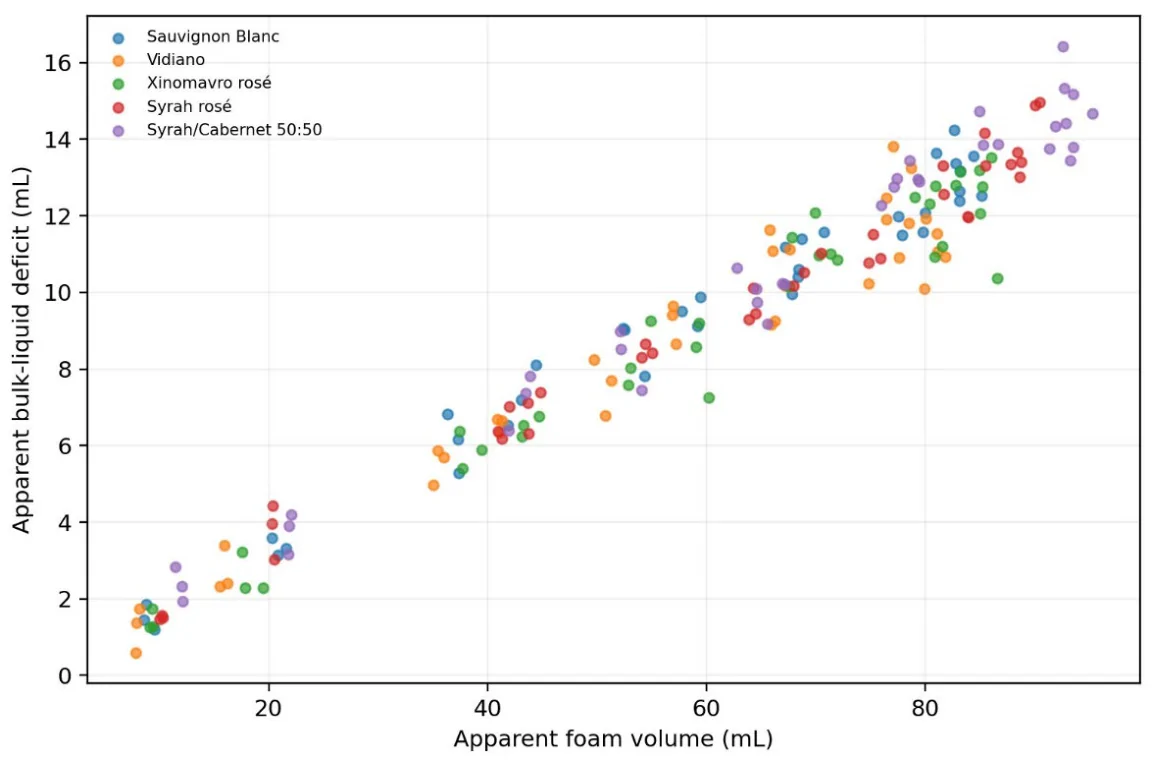
Apparent bulk-liquid deficit versus apparent foam volume for the 180 paired vessel measurements. Deficit is calculated relative to the initial 700 mL charge.

Across the 180 paired vessel measurements, the apparent bulk-liquid interface moved downward as the foam layer grew and recovered upward during the decline (Table 5; Figure 6). Pooled mean apparent foam volume increased from 9.68 mL at D0 to 86.00–86.52 mL at D10–D11, while the pooled apparent bulk-liquid mean decreased from 698.39 mL to 686.79–686.87 mL. The corresponding pooled apparent deficit therefore increased from 1.61 mL to approximately 13.1–13.2 mL around the sampled maximum.

After adjustment for categorical system and sampling day, the association coefficient was 0.113 mL apparent bulk-liquid deficit per mL apparent foam, with a vessel-clustered 95% confidence interval of 0.024–0.201. The adjusted model R^2^ was 0.968. Because both variables depend on the lower foam interface, the coefficient is interpreted as a concurrent interface-geometry association and not as a direct measure of liquid transferred into the foam phase.

### 3.5. Nominal-Freeboard Burden and Paired Net Intrusion

**Figure 7:**
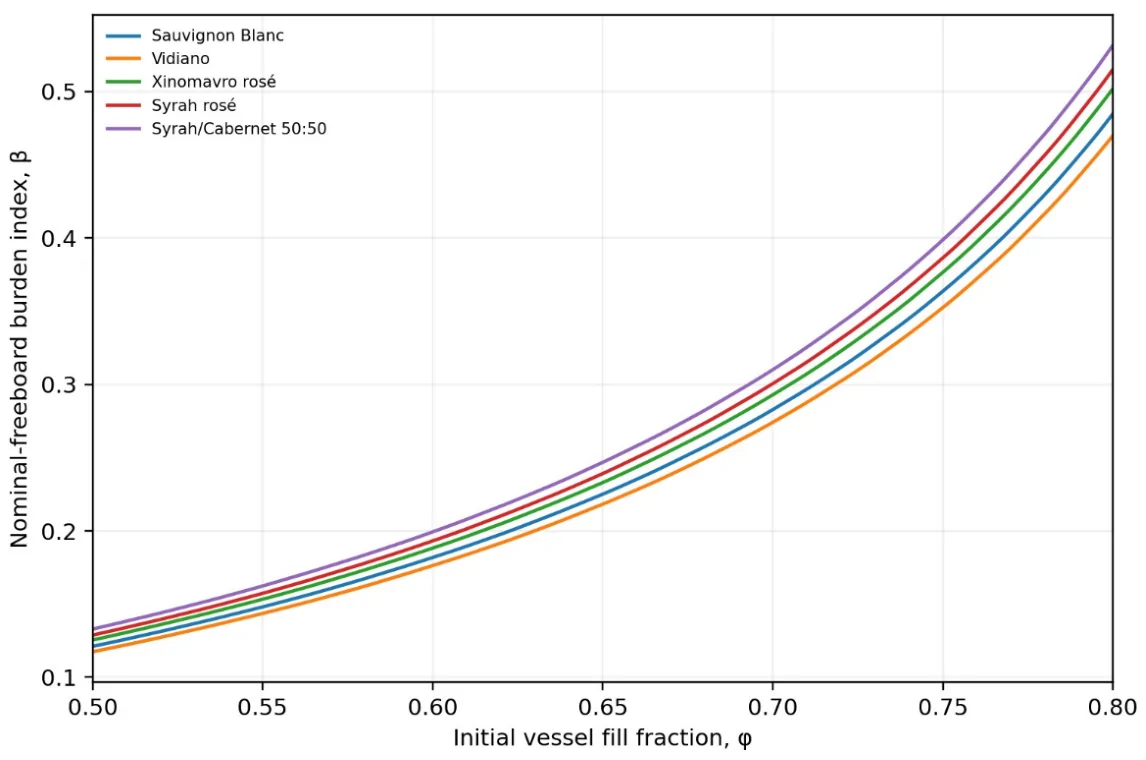
The apparent nominal-freeboard burden index calculated from each system mean *α* over the experimentally tested fill range *φ* = 0.50–0.80. The index expresses apparent foam-layer volume relative to initial nominal freeboard and should not be interpreted as a directly measured safe-fill limit.

Across the four working volumes, the mean *α* values were 12.12% for Sauvignon Blanc, 11.75% for Vidiano, 12.55% for Xinomavro rosé, 12.88% for Syrah rosé and 13.29% for the blend. At *φ* = 0.80, *β* = 4*α*, corresponding to apparent nominal-freeboard burden indices of 48.5%, 47.0%, 50.2%, 51.5% and 53.2%, respectively. Direct calculation from the measured 800 mL condition gave closely similar values of 48.33%, 46.83%, 50.17%, 52.17% and 53.17%. Figure 7 shows the geometric burden relationship only over the measured fill range *φ* = 0.50–0.80.

The paired controlled data (Table 6) illustrate the distinction between apparent foam burden and net protrusion above the initial liquid level. At each system mean sampled maximum, *β* ranged from 26.87% to 31.27% of the 300 mL nominal freeboard, whereas the paired net-intrusion fraction *γ* ranged from 23.21% to 26.25%. The difference reflects the contemporaneous downward movement of the lower foam boundary. Thus, the working-volume *β* values are best interpreted as apparent burden indices rather than direct physical occupancy of freeboard above the initial liquid surface.

### 3.6. Projected Contour Evolution

**Figure 8:**
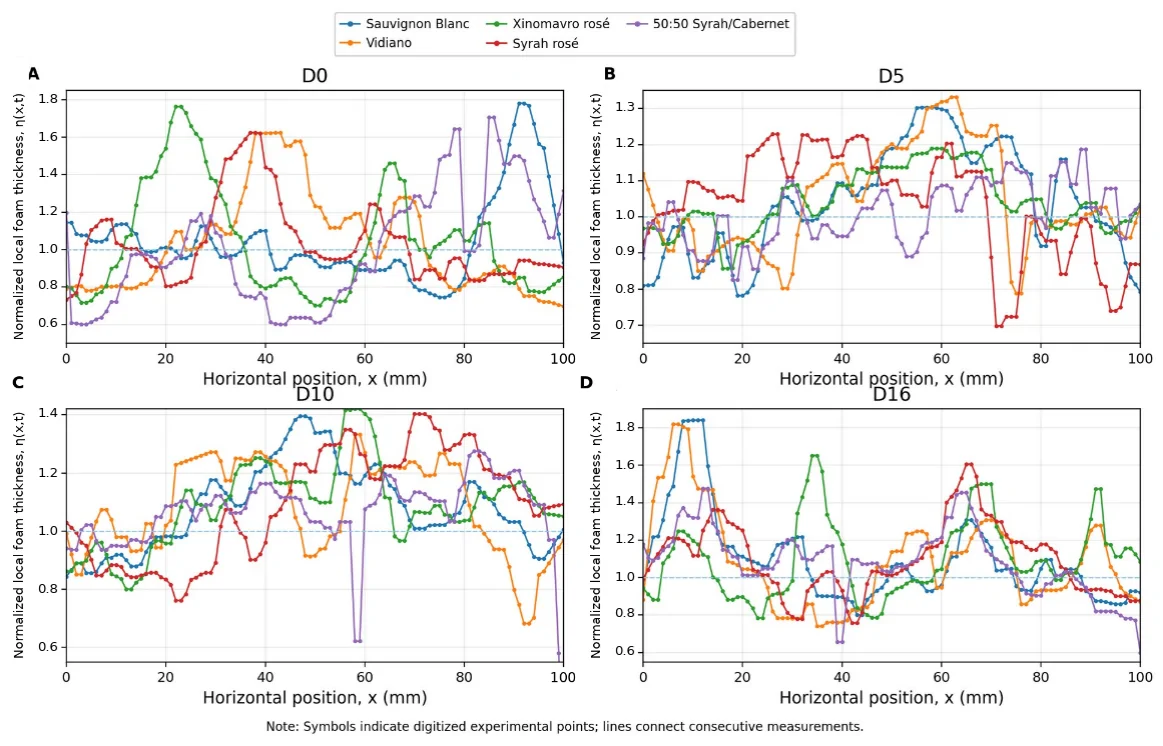
Normalized projected local foam thickness, *η*(*x*,*t*), at (**A**) D0, (**B**) D5, (**C**) D10 and (**D**) D16. The markers represent digitized contour points and the lines connect consecutive positions; the dashed horizontal reference is *η* = 1. The profiles are presented as descriptive spatial representations and were not subjected to inferential comparison among batches.

Normalized projected profiles at D0, D5, D10 and D16 showed local visible foam thickness both above and below the spatial mean (Figure 8). The distribution of peaks and troughs changed across fermentation stages and differed in appearance among the investigated batches. These profiles complement the scalar apparent-volume trajectories by showing that the visible foam layer was spatially nonuniform within the projected vessel span. Because the profiles were used as image-based descriptive representations rather than replicate-level spatial estimates, no inferential comparison was assigned to the apparent between-batch differences.

## 4. Discussion

### 4.1. Working-Volume Scaling and Nominal-Freeboard Burden

Peak apparent foam volume increased approximately proportionally with initial must charge across the investigated 500–800 mL range, while *α* remained comparatively stable. The robust sensitivity analysis did not establish a system × charge interaction, supporting an overall scaling tendency across the studied operating conditions. This finding extends earlier fermentation-foam research emphasizing the sensitivity of foam behavior to process conditions [2,28,29,30], but does not establish a scale-independent law.

Expressing apparent foam relative to the initial nominal freeboard shows why a liquid-normalized foam response of approximately 12–13% can impose a substantial burden at higher fill fractions. Here *β* relates the entire visible foam layer to initial freeboard. It must not be interpreted as direct physical occupancy above the initial liquid surface, because the lower foam boundary may also descend. This geometric distinction is examined with paired measurements in Section 4.4.

The measured scaling agrees qualitatively with the process dependence of fermentation foams described by Varley et al. [2] and with broader reports on foam control and operating conditions in bioprocesses [28,29,30]. Direct numerical equivalence is not expected, because vessel geometry, gas-release conditions, liquid composition, and operating regime differ. The present contribution is the combined, vessel-resolved reporting of apparent foam magnitude, persistence, and headspace-related descriptors in fermenting wine must.

### 4.2. Temporal Evolution and Persistence of Fermentation-Generated Foam

Peak foam volume describes the greatest sampled spatial burden, whereas AUC combines magnitude and persistence over D0–D16. The Syrah/Cabernet blend exceeded Vidiano by approximately 16.4% in controlled peak mean and 19.6% in AUC, indicating that the time-integrated difference was greater than the peak difference. This comparison retains the vessel as the experimental unit.

The Gaussian envelope succinctly summarized the dominant rise-and-decline pattern. Its high pooled R^2^ does not imply a mechanistic law or prediction beyond the measured period. Sugar depletion, CO_2_ generation, gas retention, drainage, coarsening, and film rupture may all contribute to foam trajectories [11,31,32,33,34,35,36,37]. Since time-resolved sugar, ethanol, and CO_2_ data were not collected, the separate contributions cannot be established from foam volume alone.

### 4.3. Between-Batch Differences and Matrix Context

Under the common 12.0 ± 0.3 °C longitudinal conditions, the five investigated must batches showed reproducible differences in apparent foam accumulation. These differences concern the specific homogenized batches, with three fermentation vessels per batch; they do not constitute an independently replicated comparison of grape varieties.

Previous work has associated wine-foam properties with proteins, amino acids, polysaccharides, glycoproteins, yeast-derived macromolecules, and interactions among these constituents [3,4,5,6,23,24,25,26,27]. Coelho et al. [5] and Martínez-Lapuente et al. [4] provide physicochemical context for matrix-dependent differences in foamability and persistence. Our experiments, however, followed foam emerging during active alcoholic fermentation, not foam generated in finished sparkling wine or reconstituted wine solutions. Consequently, we compare physical interpretations and methods rather than treating numerical endpoints from dissimilar protocols as interchangeable.

The measured AUC values ranged from 798.60 to 954.85 mL·day. The observed between-batch differences may reflect composition and fermentation dynamics, but those determinants were not independently measured. Chemical composition, gas-generation kinetics, and foam retention must be characterized jointly in future factorial experiments to attribute the differences to specific mechanisms.

### 4.4. Concurrent Bulk-Liquid Interface Movement

The paired measurements showed that the accumulation of visible wet foam was accompanied by a downward movement of its lower boundary, followed by a partial recovery of the apparent bulk-liquid level during foam decline. Between D11 and D16, mean apparent foam volume decreased from 86.52 to 44.96 mL while the apparent bulk-liquid reading increased from 686.87 to 692.88 mL. By D16, the apparent deficit relative to the initial 700 mL was 7.12 mL, or 1.02%. This percentage characterizes an image-defined interface displacement, not an independently measured net liquid-volume loss.

The evolution is consistent with the two-phase nature of fermentation-generated wet foam. Gas bubbles are separated by films and Plateau borders containing must-derived liquid; as foam drains and partially collapses, some of that liquid returns to the bulk while gas is released [11,18,34,35,36,37]. At the same time, fermentation converts sugars into ethanol and CO_2_, which can escape from the open vessels, changing the system mass and liquid properties. The experiment did not independently quantify CO_2_ evolution, foam liquid fraction, evaporation, or total mass loss; the observed interface evolution should therefore not be interpreted as a direct measurement of these fluxes.

This moving boundary is why *β* is larger than *γ* in the paired observations. Whereas *β* compares the whole apparent foam layer with the initial freeboard, *γ* discounts the apparent lower-boundary displacement and describes the geometrically estimated intrusion above the starting liquid level. This distinction is useful for interpreting space requirements without equating apparent foam-layer burden with literal headspace occupancy. Image-based wine-foam studies, including Condé et al. [38], offer a relevant methodological comparison, although the present measurements concern fermentation-generated rather than poured foam.

### 4.5. Projected Contour Profiles as Complementary Spatial Information

The normalized projected contours reveal local variations in apparent foam thickness that are not captured by a single centerline-derived volume. Profiles at D0, D5, D10, and D16 showed stage-dependent peaks and troughs, qualitatively consistent with the interplay of drainage, redistribution, coarsening, and wall-related effects in wet foams [11,18]. Their purpose is to supplement the scalar trajectories with spatial context; replicate-level spatial uncertainty was not estimated, so these profiles do not support inferential ranking of the five batches.

### 4.6. Experimental Scope and Industrial Implications

Interpretation of the results depends on the experimental conditions. In the working-volume series, white musts were held at approximately 12 °C and rosé musts at approximately 14 °C; system differences therefore combine matrix characteristics with the operational temperature regime. The separate longitudinal series compared all five musts at the common controlled temperature of 12.0 ± 0.3 °C. Each must was represented by one homogenized batch, so the data support within-batch vessel comparisons but not general variety-level rankings. The reported foam peaks are sampled maxima, and the independently measured repeatability of image digitization was unavailable. The naturally occurring initial soluble-solids concentrations were close to, not experimentally standardized at, 22 °Brix. The photographic acquisition cadence of the working-volume series and an independent calibration record for the reported 100 mm vessel diameter were not available; this limits assessment of transient peak capture and absolute diameter-dependent conversion uncertainty.

The controlled-temperature measurements ended at D16. Subsequent suspension of active temperature regulation allowed the must to approach ambient laboratory temperature, supporting the continuation of fermentation beyond the observation window. Accordingly, post-D16 behavior is not represented in the reported time series. Foam volume alone cannot separate sugar-consumption kinetics and CO_2_ generation from drainage, gas retention, or interfacial stability.

Although the working-volume results can inform preliminary assessments of foam demand relative to vessel capacity, transferring their numerical relationships from open 1 L containers to industrial tanks requires caution. Larger vessels differ in height-to-diameter ratio, hydrostatic depth, thermal gradients, wall effects, gas-disengagement geometry, and operating pressure or venting arrangements; these may alter bubble dynamics, liquid drainage, and foam persistence [18,39,40]. The combined descriptors *α*, *β*, and *γ* can guide pilot-scale sampling strategies and assessment of fermentation-capacity utilization, but cannot by themselves establish industrial safe-fill limits or quantify capacity savings.

Representative pilot and winery-scale trials should therefore track both foam and bulk-liquid interfaces, and where possible contemporaneous sugar consumption, CO_2_ evolution, and temperature. This would allow validation of the framework under the target operating conditions while accounting for fermentation performance and wine quality.

## 5. Conclusions

Fermentation-generated wet foam increased approximately proportionally with initial working volume across the tested 500–800 mL range, while liquid-normalized peak ratios remained comparatively stable. Under the common 12 °C longitudinal regime, the five investigated must batches showed distinct sampled trajectories and vessel-level integrated foam accumulation, with the Syrah/Cabernet blend highest, Syrah rosé intermediate, Sauvignon Blanc and Xinomavro rosé overlapping, and Vidiano lowest in AUC. These differences apply to the specific batches studied and are not evidence of general varietal ranking. Paired bulk-liquid measurements showed that growth of the apparent foam layer was accompanied by downward movement of the lower interface. Consequently, apparent foam volume relative to initial freeboard is best treated as a nominal burden index rather than literal freeboard occupancy; paired data at 700 mL yielded smaller net-intrusion fractions after accounting for interface displacement. Peak burden, temporal persistence and clearly defined vessel geometry together provide a quantitative basis for preliminary assessment of fermentation-foam space requirements and for future techno-economic evaluation of low-foaming strategies, subject to validation across independent must batches, operating temperatures and vessel scales and to the preservation of fermentation performance and wine quality.

## Figures and Tables

**Table 1:** The peak-volume loading slopes and liquid-normalized foam response. The *α* summary contains 12 vessel ratios per system across the four working volumes.

Mean *α* ± SD (%)	R^2^	Slope 95% CI	Slope per +100 mL (mL)	System
12.12 ± 0.48	0.952	10.12–13.94	12.03	Sauvignon Blanc
11.75 ± 0.46	0.958	9.80–13.21	11.51	Vidiano
12.55 ± 0.35	0.979	11.41–14.03	12.72	Xinomavro rosé
12.88 ± 0.46	0.976	12.51–15.61	14.06	Syrah rosé
13.29 ± 0.47	0.97	12.41–15.91	14.16	Syrah/Cabernet 50:50

**Table 2:** Controlled apparentfoam accumulation. The AUC is the mean ± sample SD across three vessel-level AUC values. The letters denote Tukey HSD groups; systems sharing a letter have adjusted *p* ≥ 0.05.

Group	Controlled AUC (mL·D)	Controlled Peak Mean (mL)	System
c	841.10 ± 12.40	83.8	Sauvignon Blanc
d	798.60 ± 1.89	80.6	Vidiano
c	847.45 ± 6.83	85.5	Xinomavro rosé
b	887.40 ± 8.36	89.2	Syrah rosé
a	954.85 ± 8.89	93.8	Syrah/Cabernet 50:50

**Table 3:** Mixed-design repeated-measures ANOVA for controlled apparent foam volumes. Time-related degrees of freedom are Greenhouse–Geisser-corrected (*ε* = 0.2503).

*p*	Denominator df	Numerator df	F	Effect
<0.001	10	4	139.38	System
<0.001	27.54	2.75	16,740.24	Day
<0.001	27.54	11.01	15.59	System × day

**Table 4:** The Gaussian-envelope coefficients for the controlled mean trajectories. Shared parameters: *t*_p_ = 10.804 D and *σ*_t_ = 4.318 D.

Amplitude *A_s_* (mL)	Baseline *B_s_* (mL)	System
79.34	5.17	Sauvignon Blanc
78.68	3.08	Vidiano
81.71	4.32	Xinomavro rosé
83.95	5.37	Syrah rosé
87.15	7.95	Syrah/Cabernet 50:50

**Table 5:** The pooled summaries calculated from the 180 paired vessel measurements in Appendix C. The foam and bulk-liquid values are the mean ± sample SD across the 15 vessels observed on each day; the apparent deficit is 700 mL minus the pooled mean bulk-liquid reading.

Apparent Deficit (mL)	Pooled Bulk-Liquid (mL)	Pooled Foam (mL)	Day
1.61	698.39 ± 0.52	9.68 ± 1.39	D0
3.24	696.76 ± 0.70	19.48 ± 2.27	D3
6.24	693.76 ± 0.78	39.04 ± 2.98	D5
9.38	690.62 ± 0.79	61.14 ± 3.66	D7
11.21	688.79 ± 1.09	71.98 ± 4.05	D8
12.51	687.49 ± 1.25	81.02 ± 3.96	D9
13.21	686.79 ± 1.55	86.00 ± 4.39	D10
13.13	686.87 ± 1.50	86.52 ± 4.90	D11
12.79	687.21 ± 1.06	83.10 ± 5.33	D12
11.21	688.79 ± 1.11	70.00 ± 4.67	D14
8.56	691.44 ± 0.96	55.22 ± 4.96	D15
7.12	692.88 ± 0.84	44.96 ± 4.27	D16

**Table 6:** Apparent nominal-freeboard burden *β* and paired net-intrusion descriptors. The 800 mL burden uses measured peak apparent foam divided by the 200 mL initial freeboard. The final two columns use the controlled 700 mL experiment at each system mean sampled maximum; net intrusion subtracts the paired apparent bulk-liquid deficit before division by the 300 mL initial freeboard.

Net Intrusion *γ* (%)	*β* at Controlled Peak (%)	Controlled Peak Day	*β* from Mean *α* at *φ* = 0.80 (%)	*β* at 800 mL (%)	Mean *α* (%)	System
23.67	27.93	D11	48.47	48.33	12.12	Sauvignon Blanc
23.21	26.87	D10	46.98	46.83	11.75	Vidiano
24.54	28.5	D11	50.21	50.17	12.55	Xinomavro rosé
25.06	29.73	D11	51.53	52.17	12.88	Syrah rosé
26.25	31.27	D11	53.15	53.17	13.29	Syrah/Cabernet 50:50

## Data Availability

All vessel-level working-volume peak measurements, controlled longitudinal foam readings, paired foam–bulk-liquid measurements, vessel-level AUC values and Tukey HSD comparisons are provided in Appendix A, Appendix B, Appendix C, Appendix D and Appendix E of this article. The pooled summaries in Table 5 and the paired net-intrusion descriptors in Table 6 are calculated directly from those vessel-level records. Further inquiries can be directed to the corresponding author.

## Supplementary Materials

The following supporting information can be downloaded at https://www.mdpi.com/article/10.3390/ma19214755/s1, Figure S1. Volume-based distinction between apparent foam-layer burden (*β*) and net nominal-freeboard intrusion (*γ*), illustrated using pooled D11 paired readings in the controlled 700 mL series. The schematic is not an experimental photograph.

## Abbreviations

analysis of variance	ANOVA
area under the curve	AUC
heteroscedasticity-consistent covariance estimator 3	HC3
International Organisation of Vine and Wine	OIV
root mean square error	RMSE
sample standard deviation	SD

## Appendix A. Individual Working-Volume Peak Measurements

Each row contains three separately fermented vessels. Peak apparent foam volumes and sample SD are expressed in mL.

**Table A1:** Individual working-volume peak measurements.

Mean ± SD	Vessel 3	Vessel 2	Vessel 1	°C	Initial mL	System
60.67 ± 2.52	63	61	58	12	500	Sauvignon Blanc
72.67 ± 3.51	76	73	69	12	600	Sauvignon Blanc
85.00 ± 4.00	89	85	81	12	700	Sauvignon Blanc
96.67 ± 4.51	101	97	92	12	800	Sauvignon Blanc
59.20 ± 3.18	62.1	59.7	55.8	12	500	Vidiano
70.33 ± 3.51	74	70	67	12	600	Vidiano
82.00 ± 3.00	85	82	79	12	700	Vidiano
93.67 ± 3.51	97	94	90	12	800	Vidiano
62.33 ± 2.52	65	62	60	14	500	Xinomavro rosé
75.33 ± 2.52	78	75	73	14	600	Xinomavro rosé
88.50 ± 2.50	91	88.5	86	14	700	Xinomavro rosé
100.33 ± 2.52	103	100	98	14	800	Xinomavro rosé
62.67 ± 2.52	65	63	60	14	500	Syrah rosé
76.67 ± 2.52	79	77	74	14	600	Syrah rosé
92.27 ± 3.27	95.54	92.27	89	14	700	Syrah rosé
104.33 ± 3.06	107	105	101	14	800	Syrah rosé
65.00 ± 2.00	67	65	63	14	500	Syrah/Cabernet 50:50
78.67 ± 2.52	81	79	76	14	600	Syrah/Cabernet 50:50
96.25 ± 3.00	99.25	96.25	93.25	14	700	Syrah/Cabernet 50:50
106.33 ± 3.51	110	106	103	14	800	Syrah/Cabernet 50:50

## Appendix B. Individual Controlled Foam Measurements

The same three vessels were followed across the sampling days in each system. Volumes and day-specific sample SD are in mL.

**Table A2:** Controlled apparentfoam measurements for Sauvignon Blanc.

Sample SD	Mean	Vessel 3	Vessel 2	Vessel 1	Day
0.51	9	9.58	8.82	8.6	D0
0.64	20.9	21.56	20.28	20.86	D3
0.56	37	37.34	36.35	37.31	D5
0.9	58.8	59.47	57.78	59.15	D7
1.51	69.1	70.76	67.82	68.72	D8
1.21	78.4	79.78	77.55	77.87	D9
0.87	83.4	84.39	82.74	83.07	D10
1.15	83.8	85.13	83.19	83.08	D11
1.32	81.2	82.61	81	79.99	D12
0.69	68	68.36	68.44	67.2	D14
1.11	53.1	54.38	52.52	52.4	D15
1.3	43.1	44.42	41.82	43.06	D16

**Table A3:** Controlled apparentfoam measurements for Vidiano.

Sample SD	Mean	Vessel 3	Vessel 2	Vessel 1	Day
0.2	8	8.23	7.85	7.92	D0
0.33	15.9	15.94	15.55	16.21	D3
0.47	35.5	35.98	35.47	35.05	D5
0.19	57	56.91	57.22	56.87	D7
0.71	67	67.59	67.2	66.21	D8
0.94	75.9	76.44	76.45	74.81	D9
1.05	80.6	80.07	81.81	79.92	D10
1.38	80.3	78.71	81.08	81.11	D11
0.74	77.7	77.03	77.58	78.49	D12
0.14	65.9	65.76	66.04	65.9	D14
0.78	50.6	49.76	50.73	51.31	D15
0.2	41.1	41.09	40.91	41.3	D16

**Table A4:** Controlled apparentfoam measurements for Xinomavro rosé.

Sample SD	Mean	Vessel 3	Vessel 2	Vessel 1	Day
0.12	9.3	9.16	9.4	9.34	D0
1.03	18.3	19.48	17.84	17.58	D3
1.08	38.2	39.44	37.73	37.43	D5
0.61	59.5	60.19	59.3	59.01	D7
0.85	71.2	71.96	71.36	70.28	D8
1.28	80.3	81.54	80.37	78.99	D9
1.45	84.8	86	85.21	83.19	D10
0.92	85.5	86.56	84.96	84.98	D11
1.09	81.5	80.92	80.82	82.76	D12
1.3	68.4	67.81	67.5	69.89	D14
1.14	53.6	52.83	53.06	54.91	D15
0.86	43.7	43.14	43.27	44.69	D16

**Table A5:** Controlled apparentfoam measurements for Syrah rosé.

Sample SD	Mean	Vessel 3	Vessel 2	Vessel 1	Day
0.17	10.2	10.33	10.01	10.26	D0
0.12	20.4	20.29	20.38	20.53	D3
0.51	41.4	40.93	41.95	41.32	D5
0.31	64.2	63.86	64.27	64.47	D7
0.56	75.3	75.22	75.9	74.78	D8
0.89	84.9	85.45	85.38	83.87	D9
1.12	88.8	88.57	90.02	87.81	D10
1.07	89.2	88.75	90.42	88.43	D11
1.31	82.4	81.69	83.91	81.6	D12
1.28	69.1	67.96	70.48	68.86	D14
0.5	54.5	54.39	55.05	54.06	D15
0.66	44.1	43.76	44.86	43.68	D16

**Table A6:** Controlled apparentfoam measurements for Syrah/Cabernet 50:50.

Sample SD	Mean	Vessel 3	Vessel 2	Vessel 1	Day
0.38	11.9	11.47	12.17	12.06	D0
0.16	21.9	21.84	22.08	21.78	D3
1.02	43.1	43.88	43.48	41.94	D5
1.42	66.2	66.93	67.11	64.56	D7
1.3	77.3	78.54	77.42	75.94	D8
0.89	85.6	84.92	86.61	85.27	D9
0.48	92.4	92.51	92.82	91.87	D10
1.31	93.8	92.67	95.24	93.49	D11
1.18	92.7	91.35	93.5	93.25	D12
1.28	78.6	77.12	79.41	79.27	D14
1.41	64.3	62.76	65.53	64.61	D15
1.1	52.8	52.19	54.07	52.14	D16

## Appendix C. Corresponding Foam and Bulk-Liquid Measurements

Foam and bulk-liquid readings are matched within each system, vessel and sampling day. All volumes are in mL.

**Table A7:** Paired apparent foam and bulk-liquid measurements for Sauvignon Blanc.

Bulk V3	Bulk V2	Bulk V1	Foam V3	Foam V2	Foam V1	Day
698.81	698.14	698.55	9.58	8.82	8.6	D0
696.69	696.41	696.85	21.56	20.28	20.86	D3
694.72	693.17	693.83	37.34	36.35	37.31	D5
690.11	690.48	690.87	59.47	57.78	59.15	D7
688.43	690.05	688.6	70.76	67.82	68.72	D8
688.42	688.01	688.51	79.78	77.55	77.87	D9
686.43	686.63	687.6	84.39	82.74	83.07	D10
687.48	686.82	687.36	85.13	83.19	83.08	D11
685.76	686.36	687.92	82.61	81	79.99	D12
689.6	689.39	688.82	68.36	68.44	67.2	D14
692.19	690.98	690.93	54.38	52.52	52.4	D15
691.89	693.47	692.81	44.42	41.82	43.06	D16

**Table A8:** Paired apparent foam and bulk-liquid measurements for Vidiano.

Bulk V3	Bulk V2	Bulk V1	Foam V3	Foam V2	Foam V1	Day
698.25	699.41	698.63	8.23	7.85	7.92	D0
696.61	697.67	697.6	15.94	15.55	16.21	D3
694.31	694.14	695.03	35.98	35.47	35.05	D5
690.36	691.35	690.58	56.91	57.22	56.87	D7
688.88	689.83	690.74	67.59	67.2	66.21	D8
687.52	688.1	689.76	76.44	76.45	74.81	D9
688.08	689.07	689.91	80.07	81.81	79.92	D10
686.76	688.47	688.94	78.71	81.08	81.11	D11
686.18	689.09	688.2	77.03	77.58	78.49	D12
688.36	688.91	690.84	65.76	66.04	65.9	D14
691.76	693.22	692.3	49.76	50.73	51.31	D15
693.67	693.31	693.35	41.09	40.91	41.3	D16

**Table A9:** Paired apparent foam and bulk-liquid measurements for Xinomavro rosé.

Bulk V3	Bulk V2	Bulk V1	Foam V3	Foam V2	Foam V1	Day
698.75	698.73	698.25	9.16	9.4	9.34	D0
697.71	697.71	696.78	19.48	17.84	17.58	D3
694.12	694.59	693.63	39.44	37.73	37.43	D5
692.75	690.81	691.42	60.19	59.3	59.01	D7
689.15	688.99	689.02	71.96	71.36	70.28	D8
688.8	687.69	687.51	81.54	80.37	78.99	D9
686.48	687.24	686.84	86	85.21	83.19	D10
689.63	686.8	687.93	86.56	84.96	84.98	D11
687.21	689.07	687.2	80.92	80.82	82.76	D12
688.56	689.84	687.91	67.81	67.5	69.89	D14
692.41	691.96	690.74	52.83	53.06	54.91	D15
693.77	693.46	693.24	43.14	43.27	44.69	D16

**Table A10:** Paired apparent foam and bulk-liquid measurements for Syrah rosé.

Bulk V3	Bulk V2	Bulk V1	Foam V3	Foam V2	Foam V1	Day
698.49	698.54	698.43	10.33	10.01	10.26	D0
696.04	695.57	696.98	20.29	20.38	20.53	D3
693.62	692.99	693.82	40.93	41.95	41.32	D5
690.71	689.88	690.54	63.86	64.27	64.47	D7
688.49	689.11	689.22	75.22	75.9	74.78	D8
686.69	685.83	688.02	85.45	85.38	83.87	D9
686.98	685.11	686.65	88.57	90.02	87.81	D10
686.59	685.04	686.35	88.75	90.42	88.43	D11
687.43	688.03	686.7	81.69	83.91	81.6	D12
689.83	688.98	689.48	67.96	70.48	68.86	D14
691.34	691.58	691.69	54.39	55.05	54.06	D15
693.68	692.62	692.88	43.76	44.86	43.68	D16

**Table A11:** Paired apparent foam and bulk-liquid measurements for Syrah/Cabernet 50:50.

Bulk V3	Bulk V2	Bulk V1	Foam V3	Foam V2	Foam V1	Day
697.17	698.07	697.67	11.47	12.17	12.06	D0
696.09	695.8	696.84	21.84	22.08	21.78	D3
692.19	692.64	693.61	43.88	43.48	41.94	D5
689.77	689.79	689.91	66.93	67.11	64.56	D7
686.55	687.02	687.72	78.54	77.42	75.94	D8
685.27	686.13	686.15	84.92	86.61	85.27	D9
683.57	685.58	685.66	92.51	92.82	91.87	D10
684.67	685.32	684.82	92.67	95.24	93.49	D11
686.25	686.21	686.55	91.35	93.5	93.25	D12
687.23	687.11	687.05	77.12	79.41	79.27	D14
689.36	690.82	690.25	62.76	65.53	64.61	D15
691.48	692.56	691.01	52.19	54.07	52.14	D16

## Appendix D. Vessel-Level Integrated Foam Accumulation

AUC is calculated from the complete controlled time course using the actual sampling intervals. Units are mL·D.

**Table A12:** Vessel-level integrated foam accumulation.

Mean ± SD	Vessel 3	Vessel 2	Vessel 1	System
841.10 ± 12.40	855.26	832.19	835.85	Sauvignon Blanc
798.60 ± 1.89	796.82	800.58	798.4	Vidiano
847.45 ± 6.83	855.16	842.18	845	Xinomavro rosé
887.40 ± 8.36	882.61	897.05	882.54	Syrah rosé
954.85 ± 8.89	950.16	965.1	949.29	Syrah/Cabernet 50:50

## Appendix E. Tukey HSD Comparisons for Vessel-Level AUC

Two-sided multiplicity-adjusted *p* values for all ten pairwise comparisons.

**Table A13:** Tukey HSD comparisons for vessel-level AUC.

Adjusted *p*	Mean Difference (1–2)	System 2	System 1
0.00075	42.5	Vidiano	Sauvignon Blanc
0.880551	−6.35	Xinomavro rosé	Sauvignon Blanc
0.000375	−46.30	Syrah rosé	Sauvignon Blanc
<0.000001	−113.75	Syrah/Cabernet 50:50	Sauvignon Blanc
0.000241	−48.85	Xinomavro rosé	Vidiano
0.000001	−88.80	Syrah rosé	Vidiano
<0.000001	−156.25	Syrah/Cabernet 50:50	Vidiano
0.001218	−39.95	Syrah rosé	Xinomavro rosé
<0.000001	−107.40	Syrah/Cabernet 50:50	Xinomavro rosé
0.000014	−67.45	Syrah/Cabernet 50:50	Syrah rosé
