# Supplementary material for: Fermentation-Generated Wet Foams: Working-Volume Scaling, Temporal Evolution and Nominal-Freeboard Burden
Source: Materials (Basel). 2026 Nov 9;19(21):4755. doi: 10.3390/ma19214755 (PMC13764955; doi:10.3390/ma19214755)
Supplement: Supplementary file 1 [file materials-19-04755-s001.zip › materials-4657586-supplementary.pdf]

Supplementary Material

Fermentation-Generated Wet Foams: Working-Volume Scaling, Temporal Evolution and Nominal-Freeboard Burden

Figure S1. Apparent foam burden and net freeboard intrusion

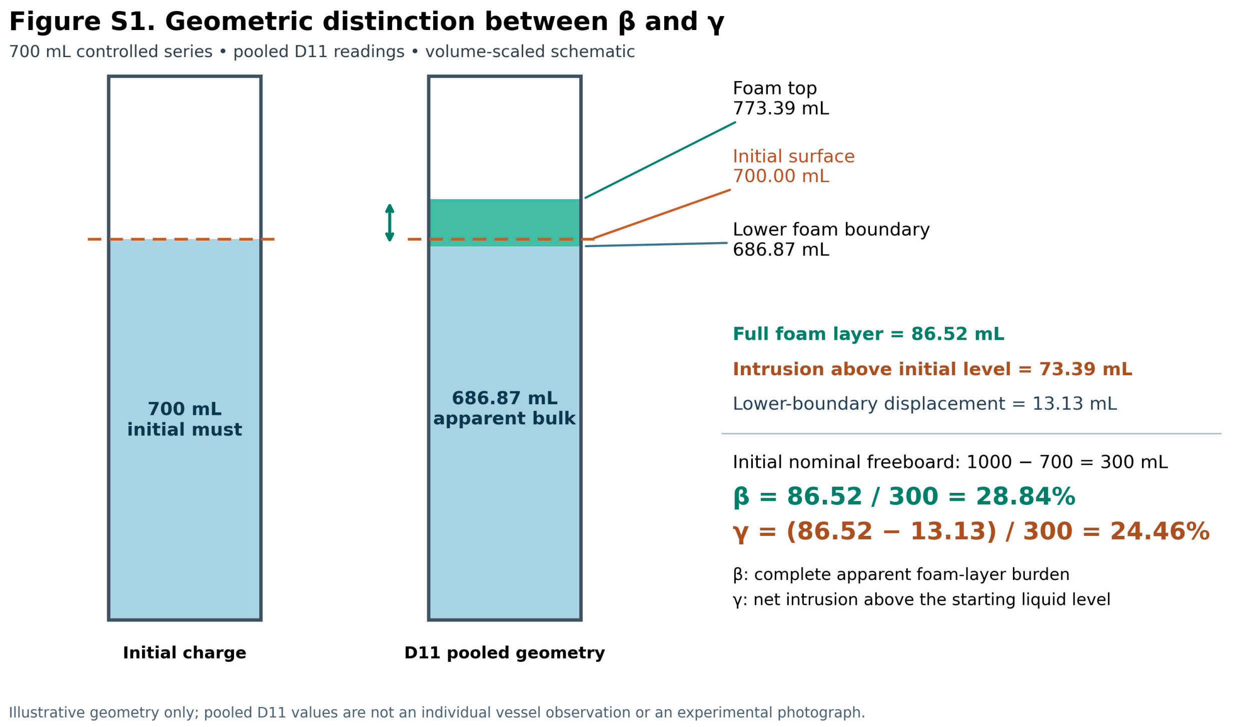

Figure S1. The apparent foam layer and its net protrusion above the initial liquid level are distinct geometric quantities. The volume-scaled schematic uses the pooled day-11 (D11) values from Table 5 of the manuscript: initial charge 700 mL, apparent foam-layer volume 86.52 mL, and apparent bulk-liquid volume 686.87 mL. The lower-boundary displacement is 13.13 mL; the estimated foam top is 773.39 mL in volumetric coordinates, with a 300 mL initial nominal freeboard, β = 28.84% and γ = 24.46%. The diagram illustrates pooled contemporaneous mean measurements, not an individual experimental vessel, an independent validation of geometric dimensions, or an actual photograph.

Definitions: $\beta = V_F/(V_T - V_{L,0})$; $\gamma = [V_F - (V_{L,0} - V_L)]/(V_T - V_{L,0})$. These quantities do not establish a safe-fill threshold or an independent measurement of liquid transferred between foam and bulk.
